# Implementation of an in situ simulation-based training adapted from Morbidity and Mortality conference cases: effect on the occurrence of adverse events—study protocol of a cluster randomised controlled trial

**DOI:** 10.1186/s13063-022-06040-2

**Published:** 2022-02-02

**Authors:** Nicolas Michel, Bernard Bui-Xuan, Lionel Bapteste, Thomas Rimmele, Marc Lilot, François Chollet, Hélène Favre, Antoine Duclos, Philippe Michel

**Affiliations:** 1 Departments of Anesthesia and Intensive Care, Hospices Civils of Lyon, Lyon, France; 2 Centre Lyonnais d’Enseignement par Simulation en Santé (CLESS, high fidelity medical simulation center), SAMSEI, Lyon, France; 3 EA 7426 “Pathophysiology of Injury-Induced Immunosuppression” (Pi3), Claude Bernard Lyon 1 University-Biomérieux-Hospices Civils of Lyon, Lyon, France; 4 Department of Quality, patient safety and patient partnership, Hospices civils de Lyon, France, Université Claude Bernard Lyon 1, Health Services and Performance Research Lab (EA 7425 HESPER), Villeurbanne, France; 5 Health Data Department, Hospices Civils de Lyon, Lyon, France; 6 Quality Safety and Customer Relationship Department, Hospices Civils de Lyon, Université Claude Bernard Lyon 1, Hesper EA 7425, F -, 69003 Lyon, France

**Keywords:** Patient safety, In situ simulation training programme, Inter-professional teamwork, Cluster randomised controlled trial, Trigger tool, Adverse event, Evaluation, Change management

## Abstract

**Background:**

Morbidity and Mortality conference provides the necessary improvement measures for patient safety. However, they are an underused resource mainly because the conclusions to be drawn from the discussion and their implications for practice are not always well integrated by inpatient care teams. We therefore propose in this study two interventions to optimise their effectiveness: a passive feedback with wide dissemination by e-mail and/or on paper of the results of the Morbidity and Mortality conference to inpatient care teams and an active feedback with in situ inter-professional simulation-training programme in which scenarios will be based on cases studied in Morbidity and Mortality conference. In the present study, we hypothesise that the greatest reduction the occurrence of adverse event will be in the active feedback arm.

**Methods:**

A cluster randomised controlled study will be performed at four study sites. The unit of randomisation is wards within the study sites. Fifteen wards will be randomly assigned to passive feedback, active feedback, or a standard MMC (control arm). Passive feedback and active feedback arms will be compared to standard arm in terms of occurrence of adverse events. The trigger tool methodology used to identify adverse events is a retrospective review of inpatient records using “triggers”: an adverse event is defined as a patient’s stay with at least one positive trigger.

**Discussion:**

The in situ simulation training based on cases processed in Morbidity and Mortality conference is built according to the main topics identified for the successful implementation of healthcare simulation in patient safety programmes: technical skills, nontechnical skills, assessment, effectiveness, and system probing. The in situ simulation-training programme conducted as part of the study has the potential to improve patient safety during hospitalisation. We therefore expect the greatest reduction in the occurrence of adverse events in patients hospitalised in the active feedback arm. This expected result would have a direct impact on patient safety and would place in situ simulation at the highest level of the Kirkpatrick model.

**Trial registration:**

Clinicaltrials.gov NCT02771613. Registered on May 12, 2016. All items from the WHO Trial Registration Data Set can be found within the protocol.

**Supplementary Information:**

The online version contains supplementary material available at 10.1186/s13063-022-06040-2.

## Background

Any human activity that presents a potential risk develops the analysis of adverse outcomes to learn from mistakes made [1]. In the medical field, Morbidity and Mortality conferences (MMC) have been used for more than 100 years, first in the USA and then around the world, to improve practice by examining medical errors and poor outcomes [2–5]. MMC is defined as a collective, retrospective, and systemic analysis of cases involving death, complication, or event that could have caused harm to the patient, with the objective of implementing actions to improve patient care [3]. MMCs provide the necessary improvement measures for patient safety and professional learning [6–10]. Nowadays, MMCs exist in many healthcare organisations but is a resource that is not used to its full potential mainly because the conclusions to be drawn from the analyses/discussions and their implications for change in practice and behaviour are not always well implemented by inpatient care teams [11, 12]. In our university hospital federation (*Hospices Civils de Lyon*, Lyon, France), despite the existence of an online procedure to prepare and standardise MMCs, two major obstacles have been identified for optimal efficiency: on the one hand, the lack of dissemination of information from return of experience, and on the other hand, the lack of effective implementation of corrective measures, most often due to the absence of a person responsible for the application and follow-up of this feedback [13]. As a result, there is a need for effective and achievable methods to improve the implementation, monitoring, and effectiveness of MMCs. The MMC Simulation project described herein (in situ simulation-based training adapted from MMC cases) tries to fill these gaps by designing a training programme using innovative methods. This is based on simulation-based teaching, an increasingly applied, evidence-based, teaching method [14]; in situ simulation-based training is defined as a simulated session in the exact environment in which it is intended to take place, as opposed to simulation centres, and which is particularly adapted for team training within an institution [15]. The latter can be an interesting method to improve care delivery in high pressure situations requiring the coordination of many people, actions, and resources; it is inter-professional and aims to raise the team’s awareness of the factors that have contributed to local events [16]. The goal is the appropriation of corrective measures*.* However, few studies have evaluated whether such interventions can improve the quality of inpatient care [17, 18] and, prior to the trial described herein, no study has evaluated the impact of these interventions on adverse events related to the care provided.

We hypothesise that the greatest reduction the occurrence of adverse event will be in the active feedback arm (in situ simulation-training programme with scenarios adapted to the clinical cases processed in MMC) than in the passive feedback arm (wide dissemination by e-mail and/or paper of the results of the MMC) and standard control arm. In the present report, we describe the methodology, and analytical plan for the MMC Simulation project.

## Methods and analysis

### Outcome and measurement tool

The outcome is the incidence rate of adverse event in relation to the trigger tool criteria. It will be calculated as the number of stays with an adverse event divided by the total number of stays in each arm during a designated period. An adverse event is defined as a patient’s stay with at least one positive trigger. Evolution of incidence rates before and during the intervention phase will be compared between the active feedback, passive feedback, and control arms.

To effectively measure adverse events, we will use a French version of the trigger tool methodology [19]. This method is easy to customise and can be easily taught; it is a retrospective review of inpatient records using “triggers” to isolate potential adverse events that provides a consistent measurement of adverse events and which is reported to provide an effective detection of adverse events [20–23]. Since 2010, this tool is accepted as valid to determine whether adverse events decrease over time as a result of improvement efforts [24]. The Institute for Healthcare Improvement (IHI) global trigger tool has been translated into French by our team. The simple nature of its clinical information did not make relevant the use of cross-language translation methods. A local trigger tool, derived from the IHI global trigger tool, was further adapted according to the specificities of each specialty by the department heads: each trigger was adapted to each specialty. The local trigger tool was constructed from a random panel of patient records by looking for “trigger elements” characteristic of adverse events that occurred in these patients. The detection of an adverse event will fully depend on the presence of any of the “trigger elements”. “Trigger elements” are based on critical criteria such as the detection of diagnostic or therapeutic complications, transfer to a higher level of care, abrupt cessation of treatment, or the use of a narcotic antagonist, for example. It is composed of clinical criteria, administrative criteria, therapeutic criteria, and biological criteria, which are easy to collect in the data and are divided into six modules: surgical module (7 items), obstetric surgery (11 items), intensive care module (9 items), and emergency care module (5 items). The local global tool with its “trigger elements” is available in the Additional file 2.

### Study design

**Fig. 1:**
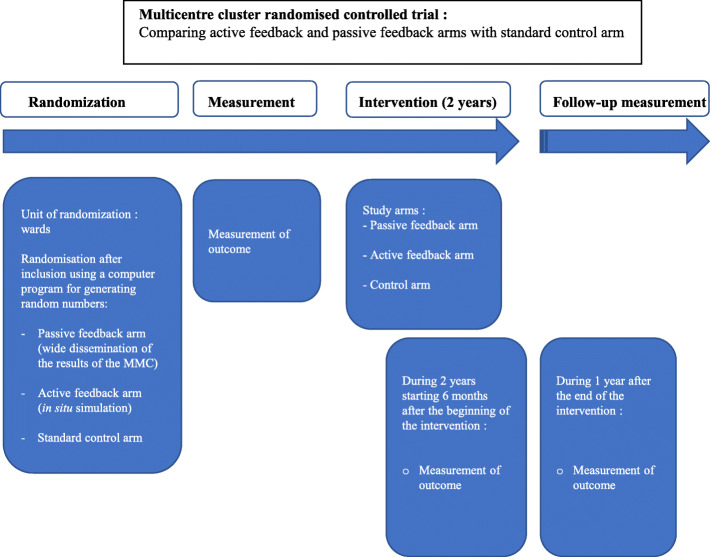
Study process

A cluster randomised controlled study constituted by three arms will be conducted. The cluster units will be wards in the participating hospitals. After agreement to participate by the medical head of the wards, the wards will be randomly assigned to passive feedback, active feedback, or standard MMC (control arm) based on computer-generated randomisation sequence with a 1:1:1 treatment allocation ratio, stratified by centre. For methodological reasons, wards of the same specialty will belong to different hospitals in order to reduce the risk of contamination. The planned duration of the intervention is 2 years. Data will be collected at three points of assessment: (1) during 1 year prior to the intervention (pre-measurement), (2) for 2 years, starting 6 months after the start of the procedure, and (3) during 1 year after the end of the intervention (follow-up measurement) in case of significant results. Adverse event rates will be compared between arms before and during the intervention following a difference-in-difference analysis. Figure 1 illustrates the study process.

### Study setting and recruitment

**Fig. 2:**
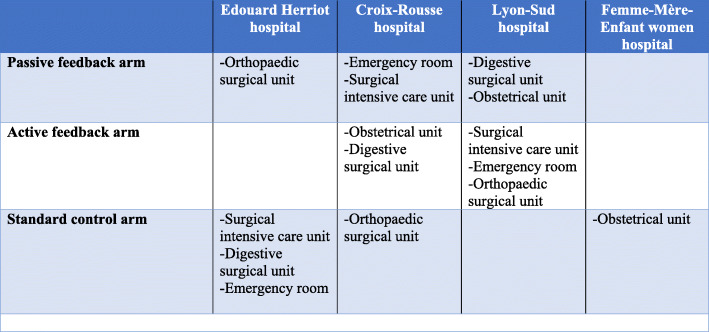
Distribution of hospital wards in the study arms

The Lyon University Hospital Federation (*Hospices Civils de Lyon* [HCL]) is a federation of academic healthcare organisations. The study concerns 15 wards among 4 hospitals (3 multidisciplinary and 1 specialised) located in Lyon (France). Figure 2 provides an overview over the submodules.

The ward specialties were chosen in accordance with the legislation of the French National Authority for Health (HAS), which has made mandatory MMC in “at risk” specialties (obstetrics, surgery, emergency, and intensive care) in the context of hospital accreditation [25]. Accordingly, surgical and trauma intensive care unit, orthopaedic surgical unit, general and digestive surgical unit, emergency room, and obstetric unit are included in the study. In those 15 wards, we will monitor outcomes among all stays in conventional hospitalisation at the exception of patients in hospitalisation by day and patients under 18 years of age.

### Sample size

#### Number of subjects expected

Over the entire study period, all stays of patients who stayed in the participating wards will be operated by the data management and analysis department (HCL) in order to measure the evaluation criteria of the trial. In total, we expect to include approximately 40,000 stays in the 1-year period before the intervention phase and 80,000 stays during the 2 years of the intervention phase.

#### Minimal effect demonstrable

Following the methodology introduced by Preisser et al. [26], we estimated the minimal demonstrable effect at a 44% reduction of adverse events in the intervention arms versus the control arm.

This estimation was based on the following assumptions: a baseline major adverse events rate of 10% for both control and intervention arms [27, 28], a post-intervention AE rate stable at 10% in the control arm, alpha risk at 5%, power at 80%, within-time ICC and between-time ICC at 0.026 and 0.022 respectively, a mean 2500 stays per ward and per year, and finally at least 40,000 stays per period (13,333 stays per arm and per period, accounting for about 5 wards).

### Intervention

As usual in the different hospitals of our institution, the MMC is structured using the guidelines of our federation to prepare and standardise the case analysis. A session should be structured to present the case, identify care problems, investigate the causes, analyse the recovery process, and finally propose an action plan. For all three study arms, at least 3 MMCs each year will be carried out in each unit during the 2 years of the intervention. Participants will not be blinded during the trial due to the nature of the intervention (passive feedback and active feedback).

For the control arm, MMCs will be organised during the intervention according to the usual format.

For passive feedback, the local coordinator in charge of MMC will choose the case analysed. A wide dissemination of information will be provided to all health professionals in the unit (medical and paramedical teams) in order to optimise the communication and increase the ownership of the professionals, in order for them to play an active role in error prevention. Therefore, the agenda of the MMC will be widely disseminated by e-mail and/or on paper, then the areas for improvement decided during the meeting, and the regular (monthly) monitoring of progress made. This monthly monitoring will check the implementation of the previous objectives with possible corrections to be made.

**Fig. 3:**
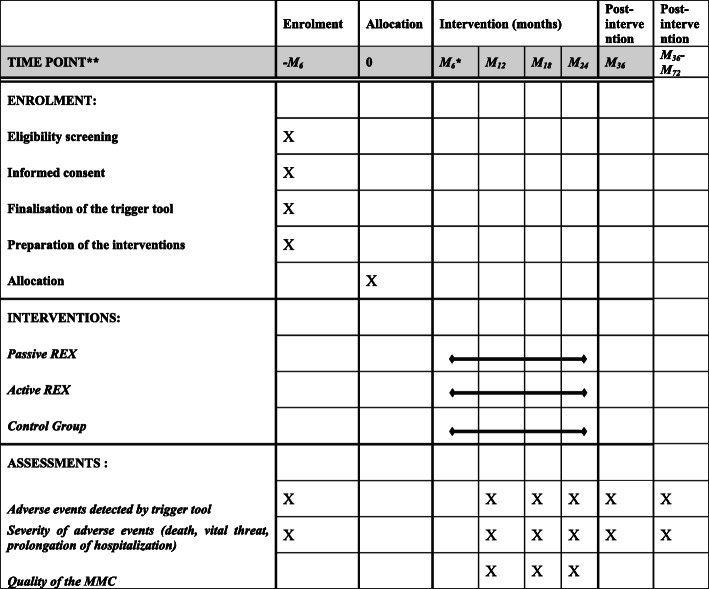
Schedule of enrolment, interventions, and assessments ((as per Standard Protocol Items: Recommendations for Interventional Trials (SPIRIT) figure)

For active feedback, it will be first suggested to broaden the choice of adverse events analysed in MMC, which is currently focused on serious events or deaths, by disseminating the list of patients with potential adverse events detected by the triggers. Then, the research group will develop an in situ simulation training programme with scenarios adapted from the cases analysed in MMC. The content of this training programme is based on the experience acquired in the simulation centre of our institution’s medical school. The in situ simulation training will be conducted in the ward during working hours in order to make the experience as close as possible to reality. This training programme is designed according to the main topics identified for the successful implementation of healthcare simulation in patient safety programmes: technical skills, nontechnical skills, assessment, effectiveness, and system probing [29]. Depending on the case to be treated, the scenario may be clinical using a patient simulator, or an interpersonal communication to learn communication skills through the simulation method. Simulation-based interpersonal communication skills training will be implemented when the adverse event is due to a communication problem: from the healthcare team to the patient, from the healthcare team to the family, or from a communication problem within the healthcare team. Depending on the case studied in MMC, the most appropriate type of simulation for each session will be discussed by the simulation team, the health services researchers of our institution, the local MMC coordinator, and the health manager of the ward concerned. After discussion between this pedagogical team, the principal investigator (BBX) will write the scenario according to the pedagogical objectives. Each simulation session will have specific objectives to promote the acquisition of appropriate technical and nontechnical skills (stress management, situational awareness, teamwork, and briefing strategies) [30]. Participants will receive a standardised briefing for each scenario. Then, an inter-professional simulation will be carried out under the responsibility of a trainer of our local centre for teaching by simulation in healthcare. The principal investigator (BBX) will be responsible for the standardised debriefing formats in order to improve situational awareness [31–33]. The debriefing will necessarily be concise with a maximum of 5 key messages for each session. The simulation sessions will be filmed and broadcast live for the entire staff present in the ward. The objective is to involve at least a quarter of the department’s staff (participants or observers). At the end of each session, participants and observers will be asked to evaluate and propose solutions to the problems identified. Figure 3 shows a schedule of study enrolment, interventions and assessments. The recommendations for interventional trials (SPIRIT) checklist is available as Additional file 1.

### Data collection and management

The data analysed will come from different sources: all the institution’s information systems, including emergency, operating room, and biology software, as well as patient records detected by the trigger tool methodology.

Our institution’s data management and analysis department (HCL) will extract these triggers from the hospital data warehouse.

The steering committee, composed by the authors, will verified the quality of the MMC, including the number and quality of participants at each meeting. It will be responsible for checking recruitment, ensuring that sites are conducting follow-ups, and checking the completeness and quality of the data. The quality of the MMC will be evaluated annually during a consensus meeting of two researchers who independently will score the quality of MMC, using a yes/no scoring system for each above-mentioned characteristic. The steering committee will meet just before database lock. This meeting will provide an opportunity to decide on issues such as possible requests for unresolved corrections, establish a baseline, and check the quality of the data. The final data set will be available upon request to the authors.

### Data analysis

The data management and analysis department (HCL) will carry out the whole process at the end of the intervention phase: data extraction and analysis will be performed by two different persons. The data manager will prepare the dataset and ensure to blind the arms of the study, so that the statistician in charge of the analysis has no clues about the arms. Analyses will only be unblinded after results obtention for final interpretation.

We will use a difference in difference (DID) approach successively to assess intervention impacts on the evolution of adverse event rates. We will compare the evolution of adverse event rates in the two intervention arms and in the control arm between the pre-measurement 1-year period and the 2-year intervention period. In case of significant differences among the arms, we may consider data collected 1 year after the end of the intervention (follow-up measurement).

Analyses will follow the intention-to-treat approach, including data from all patient’s stays. All the qualitative and quantitative variables collected will be subject to a univariate descriptive analysis. First, the variables will be described (range, interquartile range, frequency, number, mean, median, standard deviation) and compared between the intervention and control arms to ensure that there is no imbalance between them. Comparability of arms will be tested using the Rao-Scott chi-square test for qualitative variables and the Wilcoxon non-parametric test for quantitative variables to take into account the hierarchical structure of the data (multiple stays in the same ward). The power of the study as well as within-period and between-period ICC values will also be calculated.

We will then conduct multivariate analyses to model the effect of the intervention on the adverse event rate by adjusting for the arm (control, passive and active feedback), the period (before and during intervention), and arm-period interaction. We will also consider the different identified predictors or potential confounding factors. A mixed effects logistic regression model will be developed using the NLMIXED procedure to take into account the two-level hierarchical structure of the data (“stay” level nested within a “ward” level). This type of model will make it possible to measure the degree of similarity between patients of the same cluster and the variability of the endpoint between clusters. A generalised estimation equation (GEE) model approach will also be tested if relevant using the GENMOD procedure. Quantitative variables will be considered in their continuous form, except if the assumption of linearity with the endpoint studied was not verified. Possible interactions will also be sought and taken into account in the model.

All confidence intervals will be calculated at 95%, and statistical test results will be presented at the 5% threshold. Tests will be bilateral and based on non-parametric statistics if necessary. All analyses will be performed using SAS software (SAS Institute Inc., Cary, NC, USA).

### Protocol amendments

Any changes to the protocol that may affect the conduct of the study, including changes to study objectives, study design, patient population, sample sizes, study procedures, or significant administrative aspects, will require a formal amendment to the protocol and will be reported to relevant parties (e.g. trial registry, ethics committee). Important protocol modifications will be reported to relevant parties (e.g. trial registries, Ethics Committee, journals) immediately.

## Discussion

The in situ simulation training programme conducted as part of the study has the potential to improve patient safety during hospitalisation. This training programme is built according to the key factors identified for change management: a local approach (bottom-up approach) with a systemic analysis based on relevant adverse events specific to each ward, a structured implementation of MMC, and a definition by the moderator of corrective actions and their external monitoring [34]. These two interventions are of increasing complexity. The first (passive feedback) is based on actions that are simple to implement, not yet existent, but whose expected impact is limited; however, the exportability to other institutions is very good. The second (active feedback) has a more important expected effect, but its exportability is more limited to date: simulation training is nevertheless developing rapidly in other institutions, which justifies the relevance of this research project.

It is important that in situ simulations are performed on a continuous and relatively frequent basis. The minimum number of in situ simulation sessions has been set at 6 over the 2 years; this may seem modest but is justified by the technical and logistical constraints inherent in organising simulation sessions in the patient’s clinical environment. Furthermore, because of the rotation, during the same week, of medical and paramedical staff in a ward, rest days, and training days already scheduled, it seems illusory to be able to reach all, and even half of, the staff in a ward. We assumed that simulation training involving a quarter of a ward's staff would be sufficient. This teamwork will encourage active participation and allow teams to identify the factors that may contribute to local adverse events [16]. We therefore expect the greatest decrease in the occurrence of adverse events among hospitalised patients in the in situ simulation arm. This expected result would have a direct impact on patient safety and improve in situ simulation at level 4 of the Kirkpatrick model [35].

In France, the measurement of the occurrence of adverse events relies exclusively on voluntary reporting systems in healthcare facilities [36]. This imperfect method does not allow real metrological objectives. Our adaptation, in the wards concerned by this study, of the trigger tool methodology, is an important opportunity to improve knowledge of the risks associated with hospital care in France.

In conclusion, passive feedback, if effective, can very easily be implemented, with reinforcement of means, in all private or public hospitals. Active feedback can be extended to many clinical services due to the current multiplication of simulation centres in hospitals and universities. At the end of this study, we will likely be able to make recommendations to best adapt inter-professional simulation education sessions to different types of adverse events.

## Trial status

Recruitment is in progress (issue date 1 September 2020). Data collection began in July 2018 and will likely be completed in the spring of 2021.

## Dissemination

Peer-reviewed publications are planned under corporate authorship of the MMC Simulation project research team, following the Vancouver rules. We do not intend to use professional writers. The findings of the study will be communicated through a comprehensive dissemination strategy. In addition, the results of the study will also be presented at relevant national research congresses and local research symposia. The results will also be disseminated to participating departments. The final data set will be available upon request to the authors.

## Supplementary Information

**Additional file 1.** SPIRIT 2013 Checklist: Recommended items to address in a clinical trial protocol and related documents*.

**Additional file 2.** French version of the Global Trigger Tool.

## Data Availability

Not applicable.

## Abbreviations

MMC: Morbidity and Mortality conferences
RMM: Revues de Mortalité et de Morbidité
AE: Adverse event
HCL: Hospices Civils de Lyon
ALARM method: Association of Litigation and Risk Management method
HAS: Haute Autorité de Santé (French National Authority for Health)
ICC: Intracluster correlation coefficient
IHI: Institute for Healthcare Improvement
GEE: Generalised estimation equation
