# Supplementary material for: Implementation of an in situ simulation-based training adapted from Morbidity and Mortality conference cases: effect on the occurrence of adverse events—study protocol of a cluster randomised controlled trial
Source: Trials. 2022 Feb 2;23:106. doi: 10.1186/s13063-022-06040-2 (PMC8812171; doi:10.1186/s13063-022-06040-2)
Supplement: Supplementary file 2 — Additional file 2. French version of the Global Trigger Tool. [file 13063_2022_6040_MOESM2_ESM.docx]

**Additional file**

French version of the Global Trigger Tool

Surgical module:

• Indicator composed of specific indicators for each specialty

• Deep vein thrombosis or pulmonary embolism

• Surgical revision

• Cardiopulmonary arrest

• Transfer to intensive care unit

• Postoperative troponin > 300ng/L

• Decrease in hemoglobin or hematocrit by more than 25%.

Obstetrical Surgery:

• Indicator composed of specific indicators for each specialty

• Prematurity

• Cesarean section

• Death of the newborn

• Transfer to neonatal intensive care unit or intensive care (>1 day)

• Acute neonatal distress - Metabolic acidosis

• At least one transfusion of the newborn

• At least one maternal transfusion

• Third- or Fourth-degree Perineal trauma

• Postpartum hemorrhage

• Decrease in hemoglobin or hematocrit by more than 25%.

Intensive care module

• Indicator composed of specific indicators for each specialty

• SAPS II within 24 hours of admission to critical care

• Readmission to intensive care within 48 hours of discharge

• Cardiopulmonary arrest at least 24 hours after admission to intensive care

• Delivery of 3 blood bags on the same day after day 3 in ICU

• Number of organs affected increase after the 1st week

• Deep vein thrombosis or pulmonary embolism

• Hypoglycemia associated with insulin administration

• Naloxone administration

Emergency Care Module

• Indicator composed of specific indicators for each specialty

• Emergency department severity level score

• Death

• Returned home after an emergency department visit within 48 hours prior to emergency hospitalization

• Decrease in hemoglobin or hematocrit by more than 25%.
